# Development and Validation of the Immune Status Questionnaire (ISQ)

**DOI:** 10.3390/ijerph16234743

**Published:** 2019-11-27

**Authors:** Livia J. F. Wilod Versprille, Aurora J. A. E. van de Loo, Marlou Mackus, Lizanne Arnoldy, Titia A. L. Sulzer, Sterre A. Vermeulen, Smedra Abdulahad, Hendrikje Huls, Ton Baars, Andrew Scholey, Aletta D. Kraneveld, Johan Garssen, Joris C. Verster

**Affiliations:** 1 Utrecht Institute for Pharmaceutical Sciences (UIPS), Division of Pharmacology, Utrecht University, 3584 CG Utrecht, The Netherlands; l.j.f.wilodversprille@students.uu.nl (L.J.F.W.V.); a.j.a.e.vandeloo@uu.nl (A.J.A.E.v.d.L.); marloumackus@gmail.com (M.M.); l.arnoldy@students.uu.nl (L.A.); titiasulzer@gmail.com (T.A.L.S.); s.a.vermeulen@students.uu.nl (S.A.V.); s.abdulahad96@gmail.com (S.A.); ingridhuls@hotmail.com (H.H.); t.baars@fingerprint.nl (T.B.); a.d.kraneveld@uu.nl (A.D.K.); j.garssen@uu.nl (J.G.); 2 Institute for Risk Assessment Sciences (IRAS), Faculty of Veterinary Medicine, Utrecht University, 3584 CM Utrecht, The Netherlands; 3 Centre for Human Psychopharmacology, Swinburne University of Technology, Melbourne, VIC 3122, Australia; andrew@scholeylab.com; 4 Immunology Platform for Specialized Nutrition, Danone Nutricia Research, 3584 CT Utrecht, The Netherlands

**Keywords:** immune functioning, questionnaire, perceived immune status, fitness, ISQ

## Abstract

The self-assessment of perceived immune status is important, as this subjective observation leads individuals to decide whether or not to seek medical help or adapt their lifestyle. In addition, it can be used in clinical settings and research. The aim of this series of studies was to develop and validate a short questionnaire to assess perceived immune functioning. Five surveys were conducted among Dutch and International young healthy adults (18–30 years old), and two others among older age groups with various health complaints. For the first study, an existing immune functioning scale was modified and elaborated resulting in 23 immune-health-related items, of which the occurrence was rated on a 5-point Likert scale. A student sample was surveyed, and the results were used to shorten the 23-item listing into a 7-item scale with a predictive validity of 85%. Items include “sudden high fever”, “diarrhea”, “headache”, “skin problems (e.g., acne and eczema)”, “muscle and joint pain”, “common cold” and “coughing”. The scale is named Immune Status Questionnaire (ISQ), and it aims to assess perceived immune status over the preceding year. The second study revealed that the ISQ score correlated significantly with a 1-item perceived immune functioning (r = 0.383, *p* < 0.0001). In the third study, the final Likert scale descriptors were determined (“never”, “sometimes”, “regularly”, “often” and “(almost) always)”. The fourth study showed that the test–retest reliability of the ISQ is acceptable (r = 0.80). The fifth study demonstrated the association of ISQ scores with various neuropsychological and health correlates in an international sample, including perceived health and immune fitness, as well as levels of stress, fatigue, depression and anxiety. Study 6 demonstrated significant associations between ISQ scores and experiencing irritable bowel syndrome (IBS) symptoms in a sample of insomnia patients. Study 7 compared the effect of a dietary intervention in participants reporting “poor health” versus “normal health”. It is shown that ISQ scores can differentiate between those with poor and normal health, and that an effective intervention is associated with a significant improvement in ISQ scores. Data from Study 7 were further used to determine an ISQ cut-off value for reduced immune functioning, and a direct comparison with 1-item perceived immune functioning scores enabled constructing the final scoring format of the ISQ. In conclusion, the ISQ has appropriate face, content, and construct validity and is a reliable, stable and valid method to assess the past 12 month’s perceived immune status.

## 1. Introduction

An adequately functioning immune system is essential for the body to recognize and defend itself against exposure to external agents, including bacteria, viruses and substances (e.g., alcohol and drugs). Various environmental factors, lifestyle and behaviors can affect immune functioning, both positively and negatively [1]. Exposure to psychological factors (e.g., stress) can also impact immune functioning. The immune system plays an important role, either positive or negative, in various diseases and disorders and is an important health determinant [2,3,4,5,6,7,8,9,10]. For example, altered immune functioning may have a pronounced effect on normal physiological processes but is also involved in the pathology of various chronic diseases as well as certain psychiatric disorders such as depression and autism [11,12]. A combination of neuroinflammatory, neuroendocrine and metabolic effects can result in reduced immune functioning and subsequently have a negative impact on wellbeing and quality of life [6,8,13]. To identify people at risk for disease [14], assessing how well the immune system functions, i.e., immune fitness, is important to enable early intervention, for example. 

There are several ways to evaluate immune functioning. The most frequently used involve objective qualitative and quantitative assessments in blood, e.g., counts of the type and number of immune cells, immune mediators such as cytokines, chemokines and/or antibodies [14,15]. Such measures are relative costly, time-consuming and invasive. Even noninvasive assessments in saliva or urine require specialist resources and can be regarded as an imposition on those being assessed. Additionally, there is usually a time delay before results are available.

Perhaps more important, these objective assessments are often not informative about how participants experience their immune fitness, or how they feel (e.g., mood or quality of life). The latter can only be determined by subjective assessments, i.e., asking the patients how they feel. These experiential factors represent the most important determinants for participants to judge if they feel sick or healthy, and consequently seek medical help or advice [16]. These factors led us to develop a self-assessment instrument of immune fitness. This has ramifications for clinical practice, for example to determine whether further biomarker assessments are warranted. 

Such a self-assessment questionnaire has multiple potential applications, since it can be used in clinical practice, for research proposes, as well as by an individual for self-assessment. The outcome of the questionnaire is not only useful to screen for an increased risk of immune-related disease but can also influence one’s decision to seek medical attention or adapt their lifestyle. The development of such a questionnaire is essential, since a growing number of chronic diseases and disorders are linked to alterations in the immune system [17,18]. 

There are several scales developed to assess immune status, including the 1-item perceived immune functioning rating, which is used as a comparator in the studies reported here. The 1-item perceived immune functioning rating has been used in several studies as a measure of current immune fitness and showed to correlate significantly to various health outcomes, including ratings of sleep disturbance, autism, general health, mental resilience and irritable bowel syndrome (IBS) [16,19,20,21,22]. The 1-item has the advantage of being simple to administer and is currently the quickest method of determining perceived immune functioning. As a momentary assessment, however, it is by definition limited to the current perception of immune fitness. It also does not provide any information about the possible underlying cause(s) related to the outcome. 

The Immune Fitness Questionnaire (IFQ) [23] served as the basis for developing the Immune Status Questionnaire (ISQ). The IFQ does provide information about the underlying cause as it includes multiple items. A study introducing the IFQ revealed significant associations of IFQ scores with the number of general physician visits, general health and experiencing problematic internet use [23]. Although it has not been used in clinical practice, our group used the IFQ in two of our studies [19,22]. This led to the identification of certain shortcomings. Specifically, the IFQ does not capture some of the common aspects of a compromised immune function, such as muscle and joint pain or the common cold. On the other hand, it does include some relatively less common items, such as meningitis, slow healing wounds and boils.

The immune system assessment questionnaire (ISAQ) was developed as an alternative scale [24]. The ISAQ is an elaborate questionnaire with high specificity but moderate sensitivity to identify immune dysfunction. Sievers et al. [18] altered the ISAQ to obtain the infectious disease questionnaire (or “ID screen”). The ID screen is used to investigate infectious diseases and their risk factors, rather than overall immune functioning. Although it has been more extensively validated, it is not a suitable alternative for the ISQ, largely because it specifically targets identification of infectious disease rather than overall immune status. Finally, the Sickness questionnaire (SicknessQ) of Andreasson et al. [17] was developed to investigate symptoms of immune activation related to sickness behavior. Andreasson reported significant associations between SicknessQ scores and depression, anxiety, self-rated health and a single item of feeling sick. Despite the development of these questionnaires, a literature search did not identify any studies using the IFQ, ISAQ, the ID screen or the SicknessQ in clinical practice. This may be caused by the fact that the scales were elaborate and focusing on specific aspects of immune symptom functioning (e.g., infectious disease risk) rather than providing a global rating of general immune fitness assessed over a relevant period of time (e.g., the past year). Therefore, the aim of the current series of studies was to develop, validate and implement a short and cost-effective immune status questionnaire, with applicability in multiple settings, including clinical practice, research and self-assessment.

## 2. Materials and Methods 

**Figure 1:**
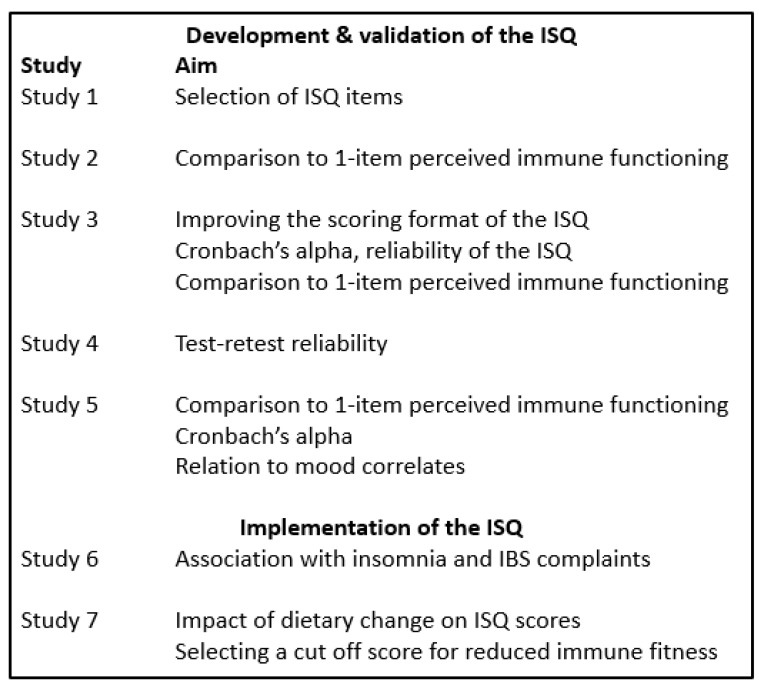
Overview of the studies.

To develop and validate the ISQ, five studies were conducted. The ISQ was implemented in two subsequent studies [25,26]. The seven studies (summarized in Figure 1) are detailed in following sections. The studies were conducted by Utrecht University and the Ethics Committee of the Faculty of Social and Behavioral Sciences of Utrecht University granted ethical approval (approval code FETC17-061).

### 2.1. Study 1: Development of the ISQ 

Study 1 aimed at developing a shortened immune functioning questionnaire compared with the IFQ. Based on discussion of the scientific literature [1] we added 4 new items to the IFQ, and it was determined which of the 23 items are relevant to predict overall immune fitness. The aim was to develop a shortened questionnaire that includes sufficient questions to have a predictive validity of at least 85% of the adapted 23-item IFQ questionnaire. The participants were Dutch students and young adults, 18 to 30 years old. They were approached via an online advertisement on Facebook, to take the online survey in SurveyMonkey. A total of *n* = 258 Dutch adults completed the survey. Their mean (SD) age was 22.9 (3.4) years old. Perceived immune functioning was assessed using the IFQ [19]. The IFQ items include “sore throat”, “headaches”, “flu”, “runny nose”, “coughing”, “cold sores”, “boils”, “mild fever”, “warts/verrucas”, ““pneumonia”, “bronchitis”, “sinusitis”, “sudden high fever”, “ear infection”, “diarrhea”, “meningitis”, “eye infection”, “sepsis” and “long healing injuries”. The four extra items were “shortness of breath”, “skin problems (e.g. acne & eczema)”, “muscle and joint pain” and “common cold”. Items were scored on a 5-point Likert scale ranging from 1 (“never”) to 5 (“frequently experienced”). The scoring of three items of the original IFQ (flu, pneumonia and meningitis) was changed from a 5-point Likert-scale to a yes/no question. Scoring of the items with a yes/no answer was 0 points for “no”. A “yes” for flu gave 2 points, while a “yes” for meningitis and pneumonia gave 4 points. The sum score of all items yielded an overall IFQ score ranging from 20 to 110, with higher scores indicating a poorer immune status. Participants were also asked to rate their current perceived immune status and perceived general health status, respectively, on a scale from 0 (very poor) to 10 (excellent) [16]. They further reported whether or not they experienced reduced immune fitness (i.e., a lowered immune functioning level compared to normal) at the moment of participation and whether or not they had a chronic disease (using a “yes”/“no” question format). 

### 2.2. Study 2: Comparison of ISQ with 1-Item Perceived Immune Functioning and General Health

Study 2 investigated the relationship between a 1-item rating of current perceived immune functioning and a 1-item rating of current general health with the shortened immune functioning questionnaire of Study 1. The participants were Dutch students and other young adults, 18 to 30 years old. They were approached via an online advertisement on Facebook, to take the online survey in SurveyMonkey. The shortened IFQ consisted of 7 representative immune associated symptoms and diseases derived from the regression analysis of Study 1 (“sudden high fever”, “diarrhea”, “headache”, “skin problems (e.g. acne & eczema)”, “muscle and joint pain”, “common cold” and “coughing”). These items were scored on a 5-point Likert scale ranging from 1 to 5 (scale ranging from “never” to “frequently experienced”). This yielded an overall immune fitness score ranging from 7 to 35, with higher scores indicating a poorer immune status. Moreover, participants were asked to complete two 1-item questions, in order to rate their perceived immune functioning and perceived general health on a scale from 0 (very poor) to 10 (excellent) [16]. They further reported whether they experienced a lowered immune functioning at the moment of participation and whether they had a chronic disease.

### 2.3. Study 3: Modifying ISQ Scoring

Study 3 investigated the effect of modifying the Likert scale for questionnaire responses in order to improve the format of the ISQ. This was based on feedback from participants of Study 1 and 2, who sometimes struggled to differentiate between responding “once or twice” and “occasionally”. Similarly, the difference between “regularly” and “frequently” was unclear to some participants. Therefore, Study 3 investigated the utility of an alternative scoring for the ISQ. Dutch students, 18–30 years old, were approached at the campus of Utrecht University, and invited to complete the survey on paper. The ISQ derived from Study 1 scored items on a 5-point Likert scale ranging from 1 to 5 with never, once or twice, occasionally, regularly and frequently experienced as anchors (overall scoring range 7 to 35, with higher scores representing poorer immune fitness). The alternative ISQ rated the same 7 immune associated symptoms and diseases in the ISQ on a 5-point Likert scale ranging from 0 to 4 with never, sometimes, regularly, often and (almost) always as anchors. Thus, the ISQ scores range from 0 to 28, with higher scores representing a poorer immune status. Again, 1-item perceived immune functioning and general health were assessed and participants indicated whether they experienced a lowered immune functioning at the moment of participation and whether they had a chronic disease. 

### 2.4. Study 4: Test–Retest Reliability of the ISQ

Study 4 investigated the test–retest reliability of the ISQ. Dutch students, 18–30 years old, were approached at the beginning of a lecture at Utrecht University, and invited to complete a short survey on paper, consisting of the ISQ and some demographic questions. Eight or ten days later the same students were approached at the beginning of another lecture and invited to complete the survey again. A total of 53 students completed the two surveys. 

### 2.5. Study 5: Exploration Health Correlates of the ISQ in an International Sample

Study 5 investigated the association of the final ISQ with two 1-item scales of perceived immune functioning, as well as various health aspects in an international young adult sample. A total of 333 young adults (18–30 years old), either at work or on holiday in Fiji, completed the ISQ. The participants originated from various countries around the world. They completed the ISQ as part of a larger study on alcohol consumption, health and immune fitness. Current perceived immune functioning and general health were rated on 1-item scales ranging from 0 (very poor) to 10 (excellent). The past year’s stress, anxiety, depression, anger/hostility, being active and fatigue were assessed on 11-point scales ranging from 0 (absent) to 10 (extreme). 

### 2.6. Study 6: Relationship of ISQ with Irritable Bowel Syndrome (IBS) Complaints in an Insomnia Sample

An online survey was conducted among 487 Dutch adults with insomnia complaints [25]. As previous research suggested a relationship between reduced immune fitness and having IBS complaints [22], in this study the ISQ was related to scores on the Birmingham IBS Symptoms Questionnaire [27]. The scale comprises subscales rating the severity of constipation, pain and diarrhea complaints. In addition, perceived current immune functioning was rated on a 1-item scale ranging from 0 (very poor) to 10 (excellent).

### 2.7. Study 7: ISQ Scores in Dutch Participants with Poor Versus Normal Health: The Impact of Dietary Change

Study 7 examined the ISQ before and after a dietary change in a Dutch adult sample with “poor health” or “normal health” [26]. A survey was conducted among Dutch adults to evaluate their health status before and after the start of consuming raw fermented milk (RFM) products [26]. They were recruited at a Dutch farm where they bought RFM products. The RFM product was labeled with an invitation flyer to complete an online survey. As part of the survey the ISQ was completed. The ISQ was completed retrospectively, assessing the participant’s immune status before and after switching to consuming RFM. In addition, 1-item perceived immune functioning was rated for the same time periods. Participants were allocated to the “poor health” group in case they reported reduced immune resistance or suffered from a chronic disease before the start of consuming RFM products. Other participants were allocated to the “normal health” group. Other variables of interest were sex and living location (rural versus urban).

### 2.8. Data Analysis

Statistical analysis was performed using SPSS (IBM Corp. Released 2013. IBM SPSS Statistics for Windows, Version 25.0. Armonk, NY, USA: IBM Corp.). For each study, the mean and standard deviation (SD) for each variable was computed. Data were examined for the whole sample, and sex differences were explored as well, using independent t-tests or Chi-squared tests. 

For study 1, a stepwise linear regression analysis was conducted to investigate which items significantly impacted the total IFQ score and should be included in the ISQ. The model should predict the IFQ score for at least 85%. For Studies 2 and 3, a Spearman correlation analysis was conducted to investigate the relationship between the ISQ and the 1-item perceived immune functioning and general health scores. In all text and tables throughout the manuscript Spearman’s rho is referred to as “r”. In Study 3, reliability analysis was conducted by computing Cronbach’s alpha, Spearman–Brown split-half, the average inter-item correlation, and the average item-total correlation. In Study 4, data from two different test occasions on which the same sample completed the ISQ were compared using nonparametric independent samples Mann–Whitney U tests, and a Spearman correlation analysis was conducted to investigate test-retest reliability. 

In Study 5, the ISQ was tested in an international sample and scores were correlated (Spearman’s rho) with the assessed health outcomes (e.g., depression and stress). In Study 6, ISQ scores were correlated with IBS outcomes by computing nonparametric Spearman’s rho correlations. Differences between men and women, between those formally diagnosed with insomnia and those with self-reported insomnia complaints, and between those reporting sleep initiation or sleep maintenance problems, or both, were computed using nonparametric independent samples Mann–Whitney U tests. In Study 7, ISQ scores were compared (1) before versus after the dietary change, (2) between men and women, (3) between participants with a “poor” and “normal” health and (4) between rural and urban living location, using nonparametric independent samples Mann–Whitney U tests. Outcomes were considered significant if *p* < 0.05. In addition, to determine a cut off point for reduced immune functioning, a direct comparison was made via linear data fitting, between ISQ scores and 1-item perceived immune functioning scores. For the 1-item perceived immune functioning scale, reduced immune fitness is assumed if a score below 5.5 is obtained. The corresponding ISQ score is proposed as ISQ cut off point for reduced immune functioning.

## 3. Results

### 3.1. Development of the ISQ (Study 1)

N = 295 participants completed the survey. The study outcomes are summarized in Table 1. 

**Figure 2:**
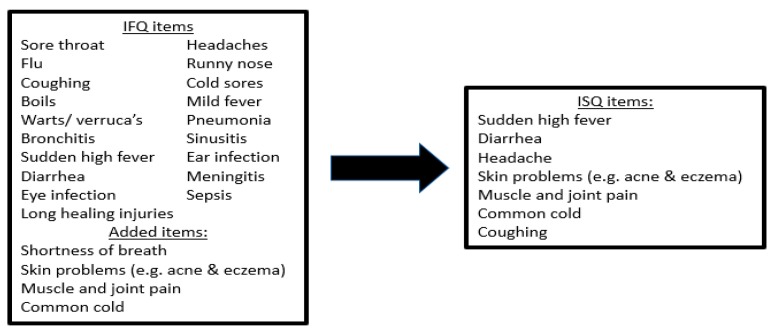
Selection of ISQ items. Regression analysis identified those items that predicted the sum score of the 23 original items for at least 85%. Abbreviations: IFQ = Immune Functioning Questionnaire, ISQ = Immune Status Questionnaire.

Significantly more women completed the survey than men. Men reported significantly better immune functioning than women. A stepwise regression analysis was performed to construct the ISQ. The total score of all 23 items was used to identify which items significantly contributed to predicting the total score (see Figure 2). These items then would form the ISQ. 

The analysis revealed that 7 items together had a predictive validity of 85.0% (see Table 2). These items were common cold, diarrhea, sudden high fever, headache, muscle and joint pain, skin problems and coughing. The seven items together formed the ISQ, used for Study 2.

### 3.2. Comparing the ISQ to 1-Item Perceived Immune Functioning and General Health (Study 2)

A total of 569 participants completed the survey. The demographics and study outcomes are summarized in Table 3. Similar to Study 1, significantly more women completed the survey than men, and men reported significantly better immune functioning than women. The ISQ had a mean (±SD) of 16.45 (±3.7). The Tukey’s Hinges 25th, 50th and 75th percentile scores were 14, 16 and 19, respectively. Correlations between the ISQ score and its individual items and 1-item perceived immune functioning and 1-item perceived general health scores are summarized in Table 4. The correlations between the ISQ and perceived 1-item immune functioning and general health were statistically significant. The item that most strongly correlated with 1-item perceived immune functioning was common cold. The strongest correlation with 1-item perceived general health was observed for the item diarrhea.

### 3.3. Improving the Scoring of the ISQ (Study 3)

A total of 291 participants completed the survey in Study 3. The demographics and study outcomes are summarized in Table 5. 

Every score from the individual items of the old and new version of the ISQ were significantly cross-correlated (*p* < 0.0001 in every case, see Table 6), suggesting that the new scoring method is adequate. 

**Figure 3:**
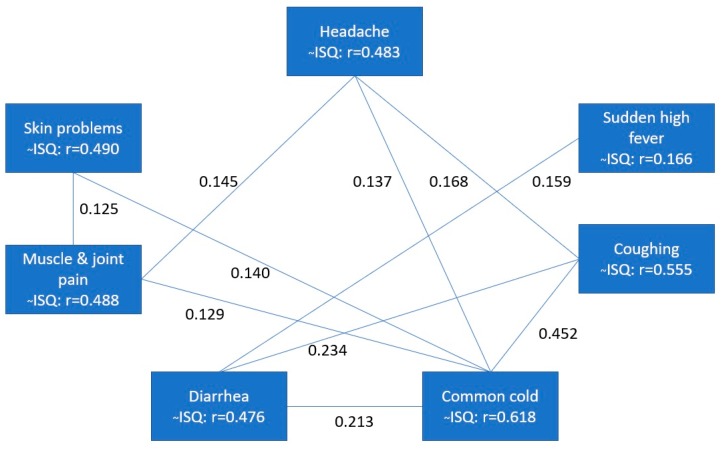
Relationship between individual ISQ items and the total ISQ score. Spearman’s rho correlations (r) were computed. Only significant correlations (*p* < 0.05) between the ISQ items are shown by connecting lines. Significant correlations of individual items with the total ISQ score (~ISQ) are also shown. Abbreviation: ISQ = immune status questionnaire.

The ISQ scores had a mean (±SD) of 7.7 (±3.1). The Tukey’s Hinges 25, 50 and 75 percentiles are 5, 7 and 10, respectively. Several tests were performed to investigate the internal consistency reliability of the ISQ. Cronbach’s alpha was 0.471, while the Spearman–Brown split-half reliability was 0.452. Furthermore, the inter-item correlations were calculated, as well as the correlations between individual item scores and the total ISQ score. These are depicted in Figure 3.

It is evident from Figure 3 that all individual items are highly and significantly correlated (r > 0.4 in all cases) with the total ISQ score. In contrast, the correlations between the single ISQ items are low (around r = 0.1) and often not statistically significant, with the exception of coughing and the common cold.

Correlations between the ISQ score and 1-item perceived immune functioning and general health are summarized in Table 7. For all items, except sudden high fever and muscle and joint pain, significant correlations were found with 1-item perceived immune functioning and general health.

### 3.4. Test Re–Test Reliability of the ISQ (Study 4)

A total of 53 participants (26.4% men and 73.6% women) completed the ISQ at two test moments, separated by 8 or 10 days. Their mean (SD) age was 18.9 (1.0) years old. A total of 37.7% of them reported reduced immune resistance and 13.2% reported having a chronic disease. Their mean (SD) 1−item perceived immune functioning and general health scores were 7.4 (1.3) and 7.6 (1.0), respectively. The mean (SD) ISQ scores on the test and re−test session were 8.4 (3.7) and 8.2 (3.6), respectively, and did not significantly differ from each other (*p* = 0.504). The test re−test reliability of the ISQ was 0.796 (*p* < 0.0001), with significant correlations for all individual items (see Table 8). 

### 3.5. Study 5: Exploration Health Correlates of the ISQ in an International Sample 

**Figure 4:**
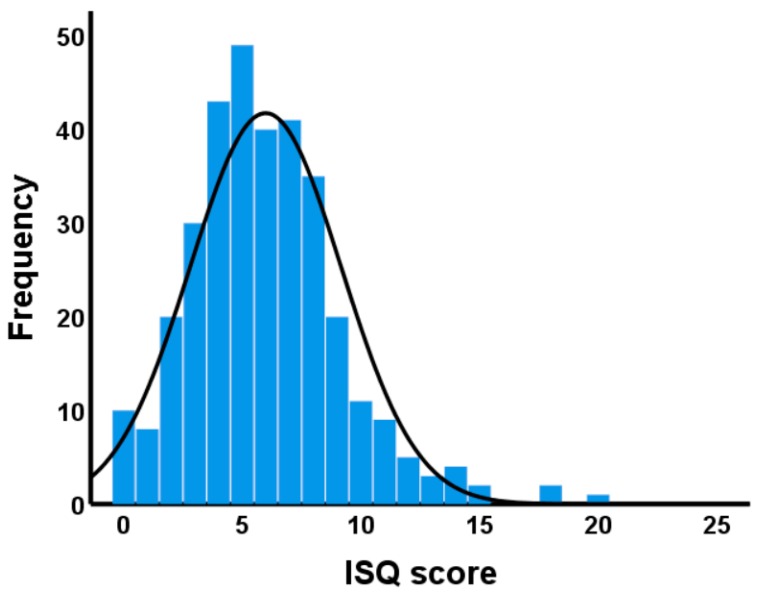
Distribution of ISQ scores. Higher ISQ scores imply poorer immune status.

A total of 333 participants completed the survey, of which 246 were on holiday and 87 worked in Fiji. The majority were residents of Australia (14.1%), Europe (73.6%) and USA (9.0%). The mean (SD) age of the sample was 24.8 (6.7) years old (56.5% were women). Their mean (SD) ISQ score was 5.8 (3.2). The distribution of ISQ scores is displayed in Figure 4 with a minimum and maximum score of 0 and 20, respectively. The Tukey’s Hinges 25, 50 and 75 percentiles are 4, 6 and 8, respectively. Cronbach’s alpha was 0.632.

No significant differences were found between participants who were in Fiji on holiday or for work, nor were any relevant sex differences observed. In this study, the ISQ correlated significantly with 1−item perceived immune functioning (r = −0.385, *p* < 0.0001) and general health (r = −0.244, *p* < 0.0001).

**Figure 5:**
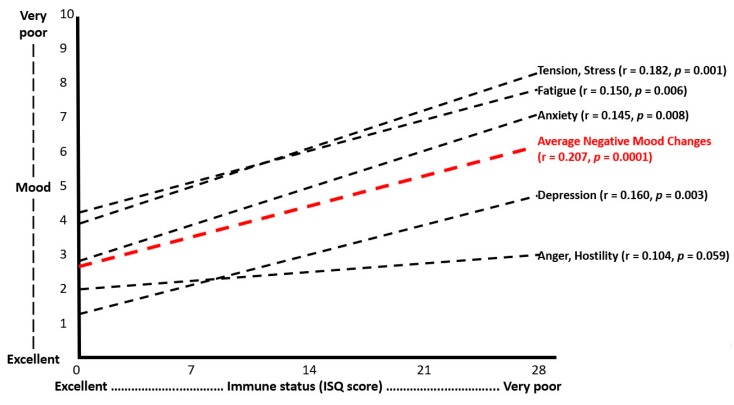
Relationship between ISQ scores and mood.

ISQ scores correlated significantly with past year’s stress, anxiety, depression and fatigue (see Figure 5). No significant correlations were found between the ISQ score and anger/hostility. The correlation between ISQ and being active was also not statistically significant.

Immune status was assessed with the immune status questionnaire (ISQ). An average “negative mood changes score” was computed by averaging the scores on the 5 mood scales. Spearman’s rho correlations (r) were considered statistically significant if *p* < 0.05.

### 3.6. Study 6: Relationship of ISQ with Irritable Bowel Syndrome in an Insomnia Sample 

In this study, 487 participants completed the ISQ. Their mean (SD) age was 37.1 (13.4) years old, and 85.3% of the sample were women. The analysis revealed that ISQ scores were significantly correlated with 1−item perceived immune functioning. ISQ scores correlated significantly (*p* < 0.0001) with the overall IBS score (r = 0.448), and the IBS subscale scores for constipation (r = 0.224), pain (r = 0.418) and diarrhea (r = 0.411). ISQ scores also correlated significantly with sleep quality (r = −0.22). ISQ scores in women (9.5 ± 9.3) were significantly higher (*p* = 0.009) than those in men (8.3 ± 3.8). ISQ scores in those formally diagnosed with insomnia (*n* = 133, ISQ = 9.8 ± 3.7) were significantly higher (*p* = 0.021) than ISQ scores in participants with self−reported insomnia complaints that were not formally diagnosed (*n* = 354, ISQ = 9.1 ± 4.0). ISQ scores also differentiated between the nature of insomnia. That is, those who report problems with both sleep initiation and sleep maintenance had significantly higher ISQ scores that those who reported only sleep maintenance problems (10.2 ± 3.9 versus 8.2 ± 3.6, respectively, *p* = 0.0001). Those who reported only sleep initiation problems had a mean (SD) ISQ score of 9.2 (4.1), which did not significantly differ from the two other groups. Detailed results of this study not pertinent to the development and validation of the ISQ are discussed elsewhere [22].

### 3.7. Study 7: ISQ Scores in Dutch Participants with Poor Versus Normal Health: The Impact of a Dietary Change

**Figure 6:**
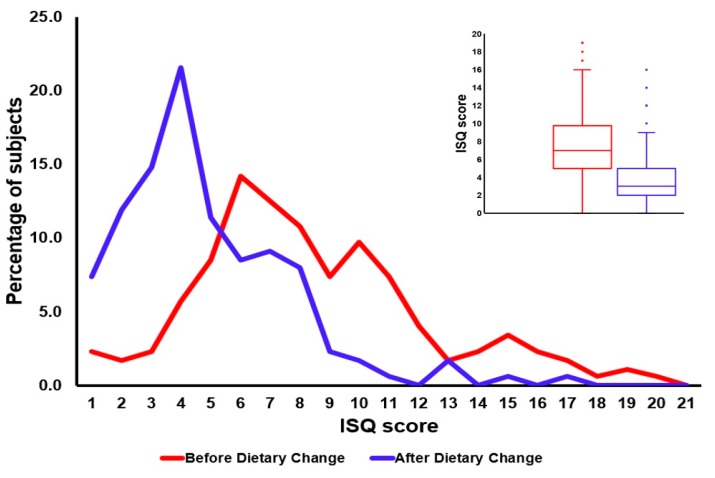
The distribution of ISQ scores before and after the dietary change to consume raw fermented milk (RFM) products. A leftward shift of the distribution indicates improved immune fitness after the dietary change. The right top box plot figure shows the minimum, first quartile (Q1), median, third quartile (Q3) and maximum ISQ scores obtained before and after the dietary change. A significant reduction (*p* < 0.0001) in mean ISQ scores was found after the dietary change, suggesting improved immune fitness (discussed in detail elsewhere [26]).

A total of 391 participants with a mean age of 54 years old completed the survey. Of the sample, 35.1% were men, and 45.0% reported to have poor health before switching to consuming RFM products. After the dietary change to RFM significant improvements on health and mood scores were reported, and the strongest improvements were reported by participants from the “poor health” group (discussed in detail elsewhere [26]). The health improvements in the “poor health” group were accompanied by a significant reduction in ISQ scores, suggesting improved immune fitness (see Figure 6).

The absolute difference in ISQ scores between the “poor health” group (ISQ = 7.4 ± 3.8) and “normal health” group (ISQ = 4.3 ± 2.7) was 3.1, and statistically significant (*p* < 0.001). In the “poor health” group, ΔISQ (ISQ before – ISQ after switching to RFM) was most pronounced (ΔISQ = −3.6, *p* < 0.001), but also the “normal health” group reported an improvement in immune fitness (ΔISQ = −1.8, *p* < 0.001). Overall, 76.0% of subjects reported an improvement in ISQ score after switching to RFM, 17.4% reported no change and 6.6% reported a worsening of immune status. Women had more health complaints before switching to RFM than men, which was reflected in significantly higher ISQ scores in women (*p* < 0.010). In both the “poor health” group and the “normal health” group sex differences in dietary change effects were small (ΔISQ < 1) and not significant. ISQ scores did not significantly differ according to living location (i.e., urban versus rural). Other results of this study, not pertinent to the development and validation of the ISQ, are discussed in the original article [26].

**Figure 7:**
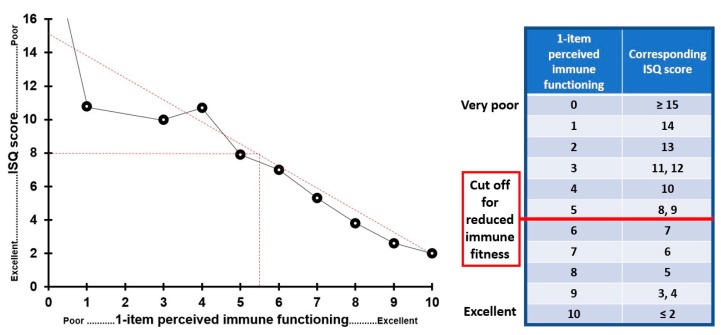
Comparison of ISQ and 1−item perceived immune functioning scores. ISQ and 1−item perceived immune functioning ratings were covering the same time period. The best fitting line through the data points is used to determine a cut off point for reduced immune functioning.

To determine an ISQ cut off score for reduced immune functioning, scores were compared with 1−item perceived immune functioning ratings that were obtained for the same time period (*n* = 902). These were scored on a scale ranging from 0 (poor) to 10 (excellent). The same scoring system is used for educational grading in The Netherlands, with a score of 5.5 as cut off for pass/fail the exam. The same cut off was used to determine a cut off for the ISQ. The results are summarized in Figure 7. The data suggest a linear relationship between ISQ and 1−item perceived immune functioning scores. It appears that a cut off for reduced immune function equals an ISQ score of 8. 

### 3.8. Final ISQ

To improve clarity, an adjustment was made to the ISQ using the time frame “past 12 months” instead of “past year” to report immune status. For example, if the survey is completed in August 2019, the time frame should be “August 2018 to August 2019”. A minority of participants, however, may interpret “past year” as “2018”. Literature confirms that in this way more precise responses are obtained for a minority of participants that interpret the time frame wrong [24]. 

To ease the understanding and interpretation of the ISQ sum score, here we propose to recalculate the original ISQ scores (score range 0 to 28) into a new 0–10 scoring format. This is accomplished using the score translation made in Figure 7, where we compared ISQ scores with a 1−point perceived immune functioning completed for the same time period (see Figure 7). Adopting the new scoring format yields a easier to interpret ISQ outcome scale. The final ISQ scoring ranges from 0 (poor) to 10 (excellent), with higher scores corresponding with better immune functioning. The final ISQ and its scoring instructions are attached as Appendix A.

The final ISQ consists of 7 items that can be scored on a Likert scale ranging from Never (0) or Sometimes (1), to Regularly (2), Often (3) and (Almost) Always (4). The ISQ score can be calculated by adding up the scores on the individual items, and subsequently recode the sum score to the 0 (poor) to 10 (excellent) scale. The proposed cut off ISQ score for reduced immune functioning is 5. 

In conjunction with the ISQ, it is advised to also assess current immune and health status (see Appendix A). Therefore, two items were added to rate (Question A) perceived immune functioning and (B) perceived general health on a scale ranging from 0 (very poor) to 10 (excellent). In addition, one question (C) asks whether participants experience perceived reduced immune fitness at this moment (to be answered “yes” or “no”), and another question (D) asks whether participant suffer from a chronic disease (to be answered “yes” or “no”). In the development of the ISQ it was not inquired what disease the participants are suffering from. It is thus unknown if the chronic disease was immune−related. In the final ISQ it is therefore asked to specify the chronic disease(s) they suffer from if this question is answered affirmative. The answer can be used to further categorize study participants.

## 4. Discussion

The ISQ is a short and practical scoring form and useful for clinical practice and research requiring a quick screening of a participant’s immune status of the past 12 months. In a series of studies, the ISQ was developed and validated. The final ISQ consists of 7 items rating past year’s immune status, by inquiring about the incidence of specific immune−related complaints. The combined scores on the items correlated significantly, albeit modest, with perceived immune fitness and several psychological corelates, such as stress and depression.

The IFQ [19] served as the basis for developing the ISQ. Based on scientific literature, several items were added. Subsequently, the new listing was shortened into the ISQ. Significant correlations of ISQ scores were found with the 1−item perceived immune functioning rating and a variety of health outcomes. The strengths of this research include the extensive validation in different studies. The ISQ is particularly useful in situations with time constraints, where the use of extensive questionnaires is not appropriate. The ISQ is also multi−applicable, since it can be used both in the clinic as for the individual as self−assessment in research surveys and screening in clinical trials. 

Across studies, even though statistically significant, the magnitude of the observed sex differences in ISQ scores is small around 1 or less on a scale ranging from 0 to 28. Therefore, the observed differences in ISQ scores between men and women do not seem to have great clinical relevance. Whereas Studies 1–5 contained primarily healthy participants, Study 7 had a larger subsample of participants reporting having a chronic disease. ISQ scores (before the dietary change) differed significantly between those with chronic diseases (i.e., “poor health” status) and healthy participants (i.e., “normal health” status).

To have a validated cut−off point at which a certain ISQ score indicates poor immune status and/or warrants further medical investigation would be of great value as it will significantly enhance the applicability of the ISQ in clinical practice. Using the original ISQ data (scoring range 0 to 28, i.e., before applying the final scoring format) suggests that a cut−off point for “poor” immune status should be 8 (see Figure 7). Indeed, the chronic disease group in Study 7 had an ISQ scores of around 8, whereas the “normal health” groups had a raw ISQ score around 4. These findings are also in line with Study 5, which showed that insomnia patients with IBS complaints had average ISQ scores ranged from 8 to 10, depending on the nature of sleep complaints. With the final scoring format of the ISQ (scoring range 0 to 10), a cut off of an “original” ISQ score of 8 corresponds to a “final” ISQ score of 5. Thus, ISQ scores < 6 are thought to indicate a poor past year’s immune status. 

It may be argued that the relatively low internal consistency, as measured with Cronbach’s alpha, is a limitation of the ISQ. However, this observation was expected and does not invalidate the ISQ. It is common that scales with a low number of unique items have a low Cronbach’s alpha [28]. This reflects the fact that immune status is a broad concept that is defined by many factors. The expression of various diseases and health complaints may contribute to it. The 7 items of the ISQ are all unique predictors of the broad concept of immune status, and correlational analysis revealed that their interrelationship is therefore low. Only the items coughing and common cold are stronger related (r = 0.452), but individually can contribute independently from each other. This can be explained by the fact that coughing is associated with a wide assortment of assortment of clinical associations and etiologies including the common cold [29]. The observed correlations in the current study between mood outcomes and ISQ were low to moderate, while at the same time these mood outcomes are often highly interrelated. This further underlines that immune fitness is a complex concept that can be independently influenced by a large number of immune−related variables. 

Finally, additional validation studies should be conducted to improve our understanding of the ISQ, and to determine how the self−rated immune functioning scores relate to objective biomarkers of immune fitness. For example, ISQ scores could be related to biomarkers of immune functioning, such as blood cytokine levels. In this context, previous research has shown significant correlations between self−rated health and immune biomarkers in both patients [30] and healthy volunteers [31,32,33]. However, another study revealed that perception of immune functioning was unrelated to immune biomarkers, including serum antibodies and blood lymphocytes [34]. Similar to our studies, the authors did find a strong relationship between perceived immune functioning and (changes in) mood. Together, up to now research investigating the relationship between objective and subjective assessments of immune functioning yielded inconsistent results. Therefore, evaluating the association of ISQ scores in relation to immune biomarkers will be the aim of future research.

## 5. Conclusions

The ISQ is a reliable, validated and short self−assessment questionnaire investigating the past 12 month’s immune status. It can be complemented by a 1−item rating of current perceived immune fitness, as suggested in our final format of the ISQ (see Appendix A).

## Figures and Tables

**Table 1:** Demographics and results for Study 1.

	**Total**	**Men**	**Women**	***p*-Value**
**Total number of participants**	*n* = 279 (100%)	*n* = 99 (35.5%)	*n* = 180 (64.5%)	0.0001 *
**Reduced immune function**	*n* = 60 (21.5%)	*n* = 11 (11.1%)	*n* = 49 (27.2%)	0.001 *
**Chronic disease**	*n* = 32 (11.5%)	*n* = 6 (6.0%)	*n* = 26 (14.4%)	0.029 *
	**Mean (SD)**	**Mean (SD)**	**Mean (SD)**	***p*-Value**
**Age (years)**	22.9 (3.4)	22.7 (3.4)	23.0 (3.4)	0.459
**Perceived general health**	8.4 (1.2)	8.5 (1.2)	8.4 (1.2)	0.249
**Perceived immune functioning**	8.6 (1.5)	8.9 (1.3)	8.4 (1.6)	0.014 *
**IFQ score**	35.8 (7.5)	33.5 (5.0)	37.1 (8.3)	0.0001 *
**ISQ score**	16.1 (3.8)	15.0 (2.9)	16.7 (4.1)	0.0001 *

*p*-values are considered significant if *p* < 0.05 (indicated by *). Abbreviations: IFQ = immune functioning questionnaire, ISQ = immune status questionnaire.

**Table 2:** Results from the regression analysis.

Model	(Added) Item	R	R Square	Item’s Predictive Validity (%)
Model 1	Common cold	0.675	0.431	43.1%
Model 2	Diarrhea	0.770	0.593	16.1%
Model 3	Sudden high fever	0.829	0.687	9.4%
Model 4	Headache	0.861	0.741	5.5%
Model 5	Muscle and joint pain	0.884	0.781	3.9%
Model 6	Skin problems (e.g., acne & eczema)	0.902	0.814	3.3%
Model 7	Coughing	0.922	0.850	3.6%

**Table 3:** Demographics and results for Study 2.

	**Total**	**Men**	**Women**	
	***n* (%)**	***n* (%)**	***n* (%)**	***p*-Value**
**Total number of participants**	*n* = 569 (100%)	*n* = 187 (32.9%)	*n* = 382 (67.1%)	0.0001 *
**Reduced immune function**	*n* = 131 (23.0%)	*n* = 27 (14.4%)	*n* = 103 (27.0%)	0.001 *
**Chronic disease**	*n* = 65 (11.4%)	*n* = 22 (11.8%)	*n* = 43 (11.3%)	0.452
	**Mean (SD)**	**Mean (SD)**	**Mean (SD)**	***p*-Value**
**Age (years)**	21.8 (3.1)	22.2 (3.2)	21.6 (3.1)	0.065
**1-item perceived general health**	7.6 (1.1)	7.6 (1.2)	7.6 (1.1)	0.719
**1-item perceived immune functioning**	7.7 (1.4)	7.9 (1.3)	7.6 (1.5)	0.004 *
**ISQ score**	16.5 (3.7)	15.3 (3.2)	17.0 (3.8)	0.0001 *

*p*-values are considered significant if *p* < 0.05 (indicated by *).

**Table 4:** Correlations between the ISQ items, perceived immune functioning and general health.

	Perceived Immune Functioning		Perceived General Health	
	Correlation	*p*-Value	Correlation	*p*-Value
**Sudden high fever**	−0.176	0.0001 *	−0.056	0.189
**Diarrhea**	−0.270	0.0001 *	−0.237	0.0001 *
**Headache**	−0.207	0.0001 *	−0.166	0.0001 *
**Skin problems**	−0.139	0.001 *	−0.131	0.002 *
**Muscle and joint pain**	−0.132	0.002 *	−0.118	0.006 *
**Common cold**	−0.328	0.0001 *	−0.082	0.055
**Coughing**	−0.243	0.0001 *	−0.128	0.003 *
**ISQ score**	−0.383	0.0001 *	−0.259	0.0001 *

*p*-values are considered significant if *p* < 0.05 (indicated by *).

**Table 5:** Demographics and results for Study 3.

	**Total**	**Men**	**Women**	
	***n* (%)**	***n* (%)**	***n* (%)**	***p*-Value**
**Total number of participants**	*n* = 291 (100%)	*n* = 88 (30.2%)	*n* = 202 (69.8%)	0.0001 *
**Reduced immune function**	*n* = 71 (24.4%)	*n* = 16 (18.2%)	*n* = 54 (26.7%)	0.077
**Chronic disease**	*n* = 32 (11.0%)	*n* = 15 (17.0%)	*n* = 17 (8.4%)	0.028 *
	**Mean (SD)**	**Mean (SD)**	**Mean (SD)**	***p*-Value**
**Age (years)**	20.8 (2.5)	21.5 (2.9)	20.4 (2.2)	0.0001 *
**1-item perceived health**	8.1 (4.7)	7.7 (1.2)	8.2 (5.5)	0.330
**1-item perceived immune functioning**	7.8 (1.3)	8.0 (1.3)	7.7 (1.3)	0.093
**ISQ old scoring**	13.4 (3.2)	13.0 (3.0)	14.4 (3.2)	0.0001 *
**ISQ new scoring**	7.7 (3.1)	6.9 (3.1)	8.0 (3.0)	0.004 *

*p*-values are considered significant if *p* < 0.05 (indicated by *).

**Table 6:** Correlation between ISQ scores with the old and new scoring.

ISQ Item	Correlation	*p*-Value
**Sudden high fever**	0.639	0.0001 *
**Diarrhea**	0.828	0.0001 *
**Headache**	0.801	0.0001 *
**Skin problems**	0.909	0.0001 *
**Muscle and joint pain**	0.840	0.0001 *
**Common cold**	0.654	0.0001 *
**Coughing**	0.704	0.0001 *
**Total ISQ score**	0.782	0.0001 *

Correlations are considered significant if *p* < 0.05, indicated with *.

**Table 7:** Spearman’s rho correlations between the ISQ scores and single items with perceived immune functioning and general health.

−−	Perceived Immune Functioning		Perceived General Health	
	Correlation	*p*-Value	Correlation	*p*-Value
**Sudden high fever**	−0.089	0.131	0.074	0.210
**Diarrhea**	−0.151	0.010 *	−0.176	0.003 *
**Headache**	−0.190	0.001 *	−0.212	0.0001 *
**Skin problems**	−0.167	0.005 *	−0.193	0.001 *
**Muscle and joint pain**	0.006	0.915	−0.062	0.295
**Common cold**	−0.341	0.0001 *	−0.226	0.0001 *
**Coughing**	−0.204	0.001 *	−0.212	0.0001 *
**ISQ score**	−0.314	0.0001 *	−0.318	0.0001 *

Correlations are considered significant if *p* < 0.05, indicated with *.

**Table 8:** Test re−test reliability of the ISQ.

ISQ Item	Correlation	*p*-Value
**Sudden high fever**	0.508	0.0001 *
**Diarrhea**	0.665	0.0001 *
**Headache**	0.827	0.0001 *
**Skin problems**	0.719	0.0001 *
**Muscle and joint pain**	0.793	0.0001 *
**Common cold**	0.758	0.0001 *
**Coughing**	0.837	0.0001 *
**Total ISQ score**	0.796	0.0001 *

Correlations are considered significant if *p* < 0.05, indicated with *.

**Figure A1:**
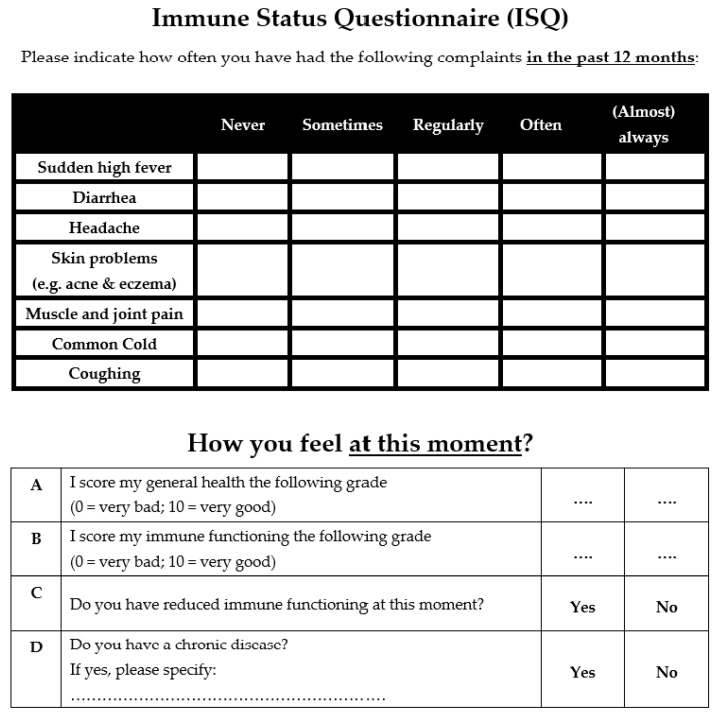
Immune Status Questionnaire (ISQ).

## Appendix A

**Table A1:** ISQ Scoring instructions.

Raw Score	Final Score
**≥15**	**0**
**14**	**1**
**13**	**2**
**11, 12**	**3**
**10**	**4**
**8, 9**	**5**
**7**	**6**
**6**	**7**
**5**	**8**
**3, 4**	**9**
**≤2**	**10**

Each item of the ISQ can be scored as follows: Never = 0 points; Sometimes = 1 point; Regularly = 2 points; Often = 3 points; (Almost) always = 4 points; Calculate the sum score of the 7 ISQ items. To obtain the final ISQ score, translate the “raw” ISQ scores as follows: Interpretation: 0 = very poor, 10 excellent perceived immune status. Cut off for reduced immune functioning: ISQ < 6.
